# Making texture descriptors invariant to blur

**DOI:** 10.1186/s13640-016-0116-7

**Published:** 2016-03-23

**Authors:** Michael Gadermayr, Andreas Uhl

**Affiliations:** Institute of Imaging and Computer Vision, RWTH Aachen University, Kopernikusstr. 16, Aachen, 52074 Germany; Department of Computer Sciences, University of Salzburg, Jakob-Haringer-Str. 2, Salzburg, 5020 Austria

**Keywords:** Texture recognition, Feature extraction, Robustness, Invariance

## Abstract

Besides a high distinctiveness, robustness (or invariance) to image degradations is very desirable for texture feature extraction methods in real-world applications. In this paper, focus is on making arbitrary texture descriptors invariant to blur which is often prevalent in real image data. From previous work, we know that most state-of-the-art texture feature extraction methods are unable to cope even with minor blur degradations if the classifier’s training stage is based on idealistic data. However, if the training set suffers similarly from the degradations, the obtained accuracies are significantly higher. Exploiting that knowledge, in this approach the level of blur of each image is increased to a certain threshold, based on the estimation of a blur measure. Experiments with synthetically degraded data show that the method is able to generate a high degree of blur invariance without loosing too much distinctiveness. Finally, we show that our method is not limited to ideal Gaussian blur.

## Introduction

For some decades, texture classification [1–16] has been a fundamental challenge in image processing. On the one hand, texture descriptors have to capture all intrinsic image properties. These are properties that contain distinctive information (for discrimination) and do not depend on the image acquisition conditions. On the other hand, extrinsic properties (i.e., properties that vary with different acquisition conditions) should not be captured, in order to maintain invariance to specific properties.

In the following, we focus on blur which is usually caused by defocus, motion, or chromatic aberrations. Although in case of good image acquisition conditions blur can mostly be prevented effectively, in many real-world scenarios, this degradation still features a present problem. One specific medical application prone to non-idealistic images is endoscopy. Firstly, it is quite difficult to adjust the distance between the lens and the surface, which is a source for defocus aberrations. Furthermore, the permanent activity of the bowel in combination with a difficult handling of the endoscope is a source for motion blur. There is significant literature on texture classification from endoscopic images such as celiac disease diagnosis [17, 18], small bowel tumor detection [19], and colon cancer detection [20].

Currently, there is only limited literature on blur-invariant texture feature extraction. Most common approaches exploit either the blur-invariant Fourier phase information of the image [4, 5] or are based on the image moment method [3]. Furthermore, previous work [21] showed that highly distinctive state-of-the-art texture features [6–9] are in general extremely sensitive to blur.

We have learned from previous work [21] that a systematic degradation, prevalent in the evaluation set, in general affects the classification accuracy by far less if the images in the training set similarly suffer from the degradation. In the referenced paper, this knowledge has been exploited by dividing the image data sets into smaller sets that are similar with respect to the level of a specific image degradation. This method is referred to as degradation adaptive texture classification. The main restriction of degradation adaptive classification is that the distribution of the degradations must be similar in the training and the evaluation set. For example, if the training set contains only idealistic images and the evaluation set contains majorly strongly degraded images, the framework does not work as there are no training images which are similar to the strongly degraded evaluation set images.

### Contribution and related work

In this work, we propose a general methodology to make image descriptors invariant to blur. Compared to degradation adaptive classification [21], such invariant texture features can be utilized more generally, as no assumptions on the distribution of the degradations in the training and the evaluation set must be made. Instead of focusing on a specific descriptor, our approach can be understood as a pre-processing technique and thereby can be applied to arbitrary texture feature extraction methods. This makes the approach highly generic as for a certain problem definition; the most appropriate state-of-the-art machine learning stage can be applied. A completely different concept is identified in the case of blur-invariant methods from literature [3–5]. These approaches apply specific concepts during feature extraction to ignore information which changes between differing blur levels.

From previous work [21], we know that blurring does not delete much distinctive information but leads to a different image representation (in the case of non-invariant feature extraction). The method proposed in this paper exploits this knowledge by equalizing the level of blur, prior to the actual feature extraction. This is done by means of an iterative algorithm based on specific blur metrics from literature.

In a comprehensive experimental evaluation, we focus on classification tasks with idealistic (blur-free) training data and distorted (blurred) evaluation data. This specific scenario is investigated because previous work [21] showed that non-invariant methods in that case are generally unable to deliver acceptable outcome. In contrast, considering classification tasks with similar degradations in the training and the evaluation set, non-invariant image descriptors have proven to be mostly quite effective [21]. Furthermore, the accuracies can be increased even more using adaptive classification. It has been shown [22] that the classification model can be adjusted effectively to partly corrupted data (during training) without noticing a strong impact on the final overall classification rates. Focusing on a scenario with idealistic training data and distorted evaluation data, these effects during classification can be eliminated to emphasize on the feature extraction stage only. Similar strategies are conducted for evaluation of scale-invariant texture descriptors [23, 24]. In real-world applications, the investigated scenario is applicable for instance if having idealistic (e.g., manually selected) training data in combination with partly degraded evaluation set data. This can be the case in specific medical applications as already mentioned above [17–20].

### Outline

This paper is structured as follows. In Section 2, the methodology of making texture image descriptors blur-invariant is introduced. In Section 3, the classification improvements are presented and discussed. Section 4 finally concludes this paper.

## Blur-invariance framework

In the following, we assume that a blurred image can be modeled by convolution of an ideal image with a Gaussian kernel. In this case, blur is referred to as Gaussian blur. The major idea of this work is to adaptively add Gaussian blur to an image to reach a specific blur level which is previously specified and is the same for all images (in the training and the evaluation set). This is done recursively by the blur-equalizing function *E*, as shown in Eq. (1):

(1) $$ E(I) = \left\{ \begin{array}{ll} I & \textrm{, if \(B(I) \geq \Theta \)} \;,\\ E(I * G) & \textrm{, if \(B(I) < \Theta \)} \end{array} \right. \;.   $$

**Fig. 1:**
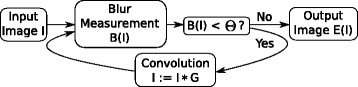
Graphical outline of blur equalization

The original image *I* is recursively convolved with a Gaussian kernel *G* until the blur measure *B* based on the image reaches the threshold *Θ*. The procedure is outlined graphically in Fig. 1.

Having perfect Gaussian blur in the images and disregarding that the desired blur strength *Θ* is not achieved exactly, the method leads to theoretically perfect invariance. For that, we exploit that a convolution of two Gaussian kernels is another Gaussian kernel and that convolution is associative. In the discrete case, besides that *Θ* is not achieved exactly, an error is furthermore obtained due to the discrete image sampling.

**Fig. 2:**
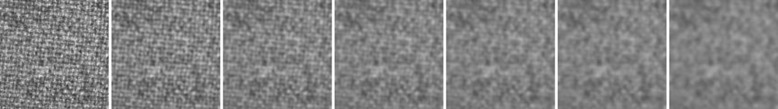
From left to right, the increased blur level during blur equalization is shown using an example texture image [27]. Between two neighboring images, exactly one convolution cycle (Fig. 1) is applied

Figure 2 shows how the blur level of an example texture image is increased during blur equalization.

### Choosing the threshold

Although the main part of the algorithm is highly simple, there remain some open issues: One question is, how to choose the threshold *Θ*. From theoretical point of view, this threshold adjusts the degree of invariance. If *Θ* is chosen to be smaller than the blur measure of some images in a database, a perfect equalization cannot be realized. Contrarily, if this value is chosen too large, the removal of high frequency information might affect the distinctiveness of the final feature vectors. To put it into a nutshell, *Θ* is the regulating parameter between high distinctiveness and a high level of invariance. In our experiments, we choose different *Θ* to investigate this correlation.

### Blur measurement

Another question is how to estimate the blur level (measured by a function *B*) in an image. For this purpose, we rely on previous work on non-reference blur measurement. We investigate two common methods [25, 26] as well as one method which does not directly estimate blur, but a related property.

- In the case of the first blur measure (*B*_Ma_) which has been introduced by Marziliano et al. [25], an edge detector is applied to find the vertical edges. Then, the local extrema are detected which correspond to the start and end points of edges. Finally, the blur metric is achieved by computing the ratio between the average edge length (i.e. the distance between start and end pixel) and the average edge magnitude (i.e. the gray value difference). A high average edge length (and/or a low edge magnitude) indicates that the edges are blurred. In opposite, short edges (and/or high edge magnitudes) means that the edges are sharp.
- Another blur measure (*B*_Cr_) has been introduced by Crete et al. [26]. In this case, the intensity variations between neighboring pixels of the original image are compared with the intensity variations of a low-pass filtered version of the image. A high variation indicates that the original image is sharp, whereas a low variation means that the original image is already blurred. Low-pass filtering is done (as proposed [26]) using an averaging filter with a size of 3 × 3 pixels.
- Finally, we will compare the two elaborated blur measurement techniques with the simple contrast property *B*_Co_ which is computed by summing up the squared differences of neighboring gray values [13] (based on horizontal and vertical neighbors). This at first sight inappropriate method is used in order to get more insight and to investigate the importance of a good blur measurement in our approach.

We investigate the behavior with these three different measures which are not built for our problem definition. Firstly, blur measures are usually not built to measure blur in textured images but rather in natural scenes. Furthermore, these metrics are constructed to measure the perceptual image degeneration and not the Gaussian *σ*.

As it is not clear, which properties of a blur measure are important in case of our scenario, in a first step, we investigate them with respect to two prediction rates. The first one (called “intra-class prediction”) measures the ability to decide which of two textures of the same class is stronger blurred with respect to the Gaussian *σ*. The second one (called “inter-class prediction”) measures the ability to decide which of two textures of different classes is stronger blurred, which is supposed to be the more difficult task. Finally, we compare these two rates with the final classification performances in order to detect correlations.

## Experiments

### Setup

**Fig. 3:**
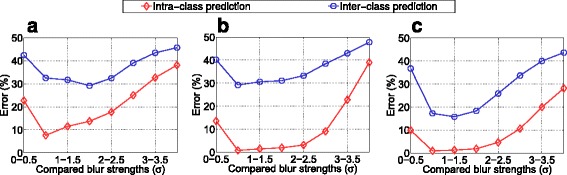
Blur measurement performances achieved with the three blur metrics

**Fig. 4:**
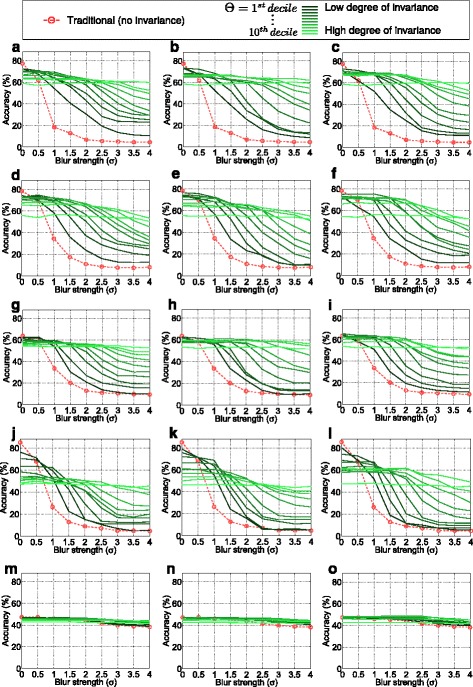
Classification accuracies achieved with different configurations

**Fig. 5:**
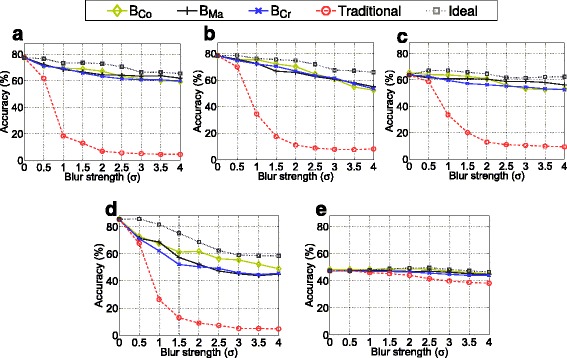
Classification accuracies if optimizing the threshold *Θ*, separately for the respective blur strength. The lines correspond to the top most points in the respective subplots in Fig. 4

**Fig. 6:**
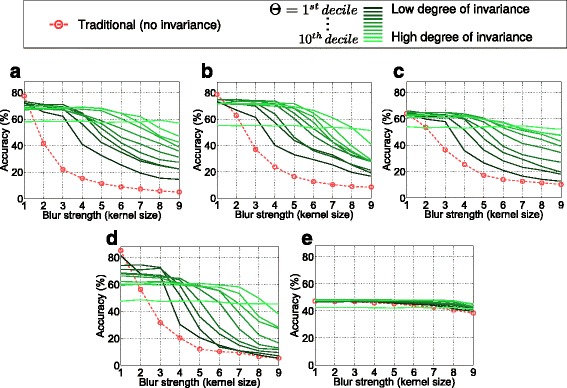
Classification accuracies with *B*_Co_, different thresholds (*Θ*) and different averaging filters (*horizontal axis*) reaching from 1×1 to 9×9 pixels

**Fig. 7:**
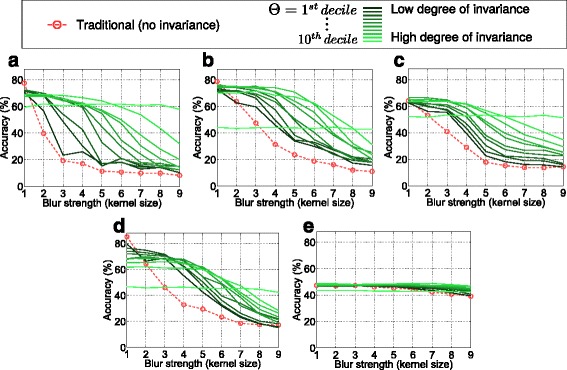
Classification accuracies with *B*_Co_, different thresholds (*Θ*) and different median filters (*horizontal axis*) reaching from 1×1 to 9×9 pixels

The experiments are based on the Kylberg texture database [27], consisting of 28 classes with 160 unique texture patches per class, captured at a single scale. Each unique database (four of them are available) contains 40 patches per class (i.e., the total number of images per set is 1120). Blurred images for the main experiments (Figs. 3, 4, and 5) are achieved by simulation, using a Gaussian kernel with varying variances *σ*. We generate images on nine different Gaussian blur levels, leading from (theoretically) *σ*=0, indicating the original image, to *σ*=4. For additional experiments (Figs. 6 and 7), images are filtered with averaging as well as median filters with sizes between 1×1 (unfiltered) and 9×9 pixels. For training, one Kylberg database (“A”) is used without any simulated blur. For evaluation, we use a separate database (“B”) with simulated blur of variable strengths (and types). For final classification, we utilize the linear support vector classifier [28]. In the experiments, the behavior with ten different blur measure thresholds (*Θ*) are investigated. They are defined by the first to the tenth ten-quantile of the blur measures in the original evaluation set (e.g., in the case of the first threshold, 10 *%* of the images are less and 90 *%* are more blurry).

For blur equalization *E* (Eq. (1)), we use a Gaussian kernel with *σ*=1 and a size of 3×3 pixels which turned out to be appropriate. Using a kernel with a larger variance, execution time can be reduced, but the accuracy might decrease slightly. A too small variance would lead to unnecessarily high computational expenses.

We do not apply any preprocessing before blur estimation. Experimentation (applying noise removal methods) did not lead to improved outcomes.

For feature extraction, five different well known techniques are investigated:

- Local binary patterns [16] (MRLBP):
  describes a texture by utilizing the joint distribution of pixel intensity differences represented by binary patterns. We deploy a multi-resolution version of the uniform patterns (capturing only patterns with at most 2 bitwise transitions) using the standard eight-neighborhood with a radius of one and two pixels. Multi-resolution in this case means that the feature vector for LBP with a radius of one and LBP with a radius of two pixels are concatenated. Due to its high distinctiveness, this feature is commonly used, although it is highly sensitive to blur [29].
- Extended local binary patterns (ELBP) [30]:
  ELBP is an edge-based derivative of local binary patterns. This descriptor is utilized with eight neighbors and a radius of one pixel. This feature is known to be similarly distinctive compared to LBP but slightly more robust to blur [29].
- Multi-fractal spectrum [7] (FRA):
  The local fractal dimension is computed for each pixel using three different types of measures for computing the local density. The feature vector is built by concatenation of these fractal dimensions. This feature is investigated because of its high discriminative power.
- Edge co-occurrence matrix [9] (ECM):
  After applying eight differently orientated directional filters, the orientation is determined for each pixel, followed by masking out pixels with a gradient magnitude below some threshold *t*. Finally, the ECM is achieved by computing the gray-level co-occurrence matrix of these data and a specified displacement *v*. For the experiments, *t* is set to 25 *%* of the maximum response and the displacement vector *v*=(1,1) is used. This feature is investigated as it could be, in opposite to the others, interpreted as a shape feature.
- Local phase quantization (LPQ) [5]:
  LPQ is based on the short-term Fourier transform, computed over a square local neighborhood. As it extracts (low frequency) phase information, this feature is declared to be robust to image blur. We choose a local neighborhood of 15×15 pixels, which turned out to be appropriate for our problem definition.

### Results

In a first experiment, focus is on the blur measures if being applied to the image textures. We would like to find out how effective Gaussian blur can be determined in an intra-class as well as in an inter-class sense (as defined in Section 2). The blur step between two compared textures in both cases is fixed to *σ*=0.5. In Fig. 3, the computed prediction errors for these two cases are presented, separately for the compared blur strengths (shown on the horizontal axes) and for all blur measures.

We notice that especially in combination with strongly blurred images, the dedicated blur measures *B*_Cr_ and *B*_Ma_ are unable (50 *%* error is achieved with guessing) to effectively measure the applied blur in an inter-class sense. Interestingly, the rather simple contrast measure *B*_Co_ seems to be even slightly more accurate in case of our problem definition. Intra-class prediction is (as supposed) easier and the measures *B*_Ma_ and *B*_Co_ are similarly competitive. *B*_Cr_ exhibits the highest error rates. Comparing images with stronger blur (e.g., images with *σ*=3 and *σ*=3.5) in general is more difficult, which is obvious as these images are more similar as far as perception is concerned. We suppose that an accurate intra-class prediction necessarily is required for our problem definition, because we assume that images of the same class should be similarly blurred in order to achieve a small feature distance. However, we do not know if an accurate inter-class prediction is necessary. Therefore, now we will focus on the final classification accuracies, achieved with the new approach, applying Eq. (1) to all images, and the different metrics.

In Fig. 4, the main results are presented. Training is done using original (non-blurred) images and evaluation is done with different Gaussian blur levels. For varying blur strengths in the evaluation set (horizontal axes), one subplot shows the traditional classification accuracies (dotted line) as well as the rates achieved with our approach. The solid lines indicate the rates obtained with our approach in combination with varying thresholds where a dark line indicates a small threshold and vice versa. First, we notice that all features, apart from LPQ, are highly sensitive to blur changes considering the traditional classification case without our pre-processing stage. Even with a small blur strength (e.g., *σ*=1), the rates drop significantly. Considering the different solid lines, it can be seen that a certain degree of invariance definitely can be achieved with our new pre-processing technique. The invariance is (as supposed) more distinct with larger thresholds *Θ*. But (also as supposed) the high degree of invariance in case of large thresholds faces lower accuracies in case of optimal images without blur. Consequently, in the case of our pre-processing method, obviously, distinctiveness has to be sacrificed for the gained invariance. However, this is not a big surprise as we know that adding significant blur slightly decreases the distinctiveness of features [21]. It is much more interesting that the distinctiveness obviously does not strongly decrease in most cases. Especially, if considering MRLBP, ELBP, or ECM, the accuracies remain quite stable with increasing thresholds, especially if compared with the blur-invariant LPQ method. However, we notice that *Θ* which regulates the invariance should be carefully chosen depending on the expected blur strengths as mostly distinctiveness must be sacrificed for a higher degree of invariance.

In Fig. 5, for each feature extraction method, each blur metric and each blur strength, the accuracies achieved with the optimal *Θ* for the specific blur strengths are plotted and compared with the classification rates achieved with traditional classification. Thereby, the blur measures can be effectively compared with each other. Additionally, we show the (“ideal”) accuracies that could be achieved if the blur measure would be able to exactly determine the Gaussian *σ*. These rates cannot be achieved in a real-world scenario (as the real Gaussian *σ* is unknown); however, they inform us about the effectiveness of the utilized blur metrics. Quite interestingly, we notice that *B*_Co_ works very effectively in case of moderate degradations (i.e., *σ* between 1 and 2) and is even able to outperform the dedicated blur metrics *B*_Ma_ and *B*_Cr_. Especially in combination with the MFS feature, the contrast measure seems to be highly competitive. This is quite astonishing, as the *B*_Co_ measure definitely is not built for blur measurement. However, our analysis (Fig. 3) already showed that it might work for our problem definition considering intra-class and inter-class blur prediction performance. Obviously, the sophisticated perceptual blur metrics are not optimally suited. Considering stronger blur, apart from the MFS feature, *B*_Ma_ is the best or at least highly competitive. Considering the plots in Fig. 3, it could be deduced that a good intra-class blur prediction is decisive (as *B*_Cr_ in general seems to be less appropriate). Moreover, a good inter-class prediction seems to be also important if considering *B*_Co_ which works effectively (and corresponds to a good inter-class prediction rate) in the lower blur range. We suppose that a good inter-class prediction is important to limit the required threshold *Θ*. If some textures correspond to outlying blur estimations, the threshold must be set high in order to achieve a certain degree of invariance. Regarding the “ideal” lines, it can be seen that the best blur measures are mostly quite competitive. Especially in case of MRLBP, ECM, and LPQ, the gap is mostly lower than 5 %. However, considering the MFS subplot, we notice that the new approach could profit even more from a more appropriate blur metric.

Finally, we investigate the effect of our pre-processing approach in case of “non-Gaussian” blur. As our method (see Eq. (1)) is based on the assumption that a blurred image is obtained by convolution of an ideal image with a Gaussian kernel, it could be supposed that the prevalence of a different kind of blur corrupts the invariance. Therefore, in Figs. 6 and 7, the same experiment as in Fig. 4 with averaging and median filtered images (the filter size is given on the horizontal axis) and the probably most appropriate blur metric *B*_Co_ are presented. We notice that the impact of averaging filtered images (Fig. 6) instead of Gaussian-filtered images is quite small. With all features, similarly good compromises between a high invariance and a high distinctiveness can be obtained. The performances of traditional classification can be outperformed significantly in each case. Especially until a size of 4×4 pixels, the loss of accuracy compared to idealistic images (1×1) is in general negligible. Is has to be mentioned that the curves cannot be directly compared to the curves in Fig. 4, because the degradation is different and thereby the traditional classification rates (dashed lines) are also different.

Considering the classification rates in case of median filtered images (see Fig. 7), it can be seen that the significantly different kind of blur (compared to Gaussian blur) actually has an impact on the invariance. Especially in the range of large kernel sizes (above 5×5 pixels) combined with lower thresholds, accuracy increases are lower compared to Gaussian or averaging filtered images. This is supposed to be due to the different properties between Gaussian blur and blur due to median filtering. However, if considering lower blur levels (up to a kernel size of 5×5 pixels), which are probably more relevant in practice, the level of invariance is still worthwhile. From these results, it can be concluded that our method is definitely not limited to image databases suffering from Gaussian blur.

## Conclusions

We have proposed a generic approach to make texture features invariant to blur. By equalizing the blur level, a high degree of invariance can be achieved without losing too much distinctiveness, which is of high relevance for practical usage. Even the robustness of a dedicated blur-robust descriptor can be improved furthermore. With all of the three tested blur measures, competitive results can be obtained. However, depending on the respective feature extraction method and the blur strength, either the contrast-based *B*_Co_ mostly in case of lower blur strengths or the more elaborated technique *B*_Ma_ leads to the best compromise between accuracy and invariance. Furthermore, we showed that the performance could be improved again if more appropriate blur measures would be available. Finally, it has been proven that although it assumes Gaussian-blurred images, our method can be successfully applied even in case of other kinds of blur.

## References

[1] Liu L, Fieguth P, Kuang G, Zha H (2011). Sorted random projections for robust texture classification. *Proceedings of the IEEE International Conference on Computer Vision (ICCV’11)*.

[2] Sifre L, Mallat S (2013). Rotation, scaling and deformation invariant scattering for texture discrimination. *Proceedings of the IEEE Conference on Computer Vision and Pattern Recognition (CVPR’13)*.

[3] Liu J, Zhang T (2005). Recognition of the blurred image by complex moment invariants. Pattern Recogn. Lett.

[4] Saipullah KM, Kim DH (2012). A robust texture feature extraction using the localized angular phase. Multimed. Tools Appl.

[5] Ojansivu V, Heikkilä J (2008). Blur insensitive texture classification using local phase quantization. *Proceedings of the International Conference on Image and Signal Processing (ICISP’08)*.

[6] Ojala T, Pietikäinen M, Mäenpää T (2002). Multiresolution gray-scale and rotation invariant texture classification with local binary patterns. IEEE Trans. Pattern. Anal. Mach. Intell.

[7] Xu Y, Ji H, Fermüller C (2009). Viewpoint invariant texture description using fractal analysis. Int. J. Comput. Vis.

[8] Dalal N, Triggs B (2005). Histograms of oriented gradients for human detection. *Proceedings of the IEEE Conference on Computer Vision and Pattern Recognition, (CVPR’05)*.

[9] Rautkorpi R, Iivarinen J (2004). A novel shape feature for image classification and retrieval. *Proceedings of the International Conference on Image Analysis and Recognition (ICIAR’04)*.

[10] Jegou H, Douze M, Schmid C, Perez P (2010). Aggregating local descriptors into a compact image representation. *Proceedings of the IEEE Conference on Computer Vision and Pattern Recognition (CVPR’10)*.

[11] Perronnin F, Liu Y, Sanchez J, Poirier H (2010). Large-scale image retrieval with compressed Fisher vectors. *Proceedings of the IEEE Conference on Computer Vision and Pattern Recognition (CVPR’10)*.

[12] Sánchez J, Perronnin F, Mensink T, Verbeek JJ (2013). Image classification with the Fisher vector: Theory and practice. Int. J. Comput. Vis. (IJCV).

[13] Haralick RM, Dinstein I, Shanmugam K (1973). Textural features for image classification. IEEE Trans. Syst. Man Cybern.

[14] Varma M, Zisserman A (2009). A statistical approach to material classification using image patch exemplars. IEEE Trans. Pattern. Anal. Mach. Intell. (TPAMI).

[15] Cimpoi M, Maji S, Kokkinos I, Mohamed S, Vedaldi A (2014). Describing textures in the wild. *Proceedings of the IEEE Conference on Computer Vision and Pattern Recognition (CVPR’14)*.

[16] Ojala T, Pietikäinen M, Harwood D (1996). A comparative study of texture measures with classification based on feature distributions. Pattern Recogn.

[17] Vécsei A, Fuhrmann T, Liedlgruber M, Brunauer L, Payer H, Uhl A (2009). Automated classification of duodenal imagery in celiac disease using evolved fourier feature vectors. Comput. Methods Prog. Biomed.

[18] Ciaccio EJ, Tennyson CA, Lewis SK, Krishnareddy S, Bhagat G, Green P (2010). Distinguishing patients with celiac disease by quantitative analysis of videocapsule endoscopy images. Comput. Methods Programs Biomed.

[19] Barbosa DJC, Ramos J, Lima CS (2008). Detection of small bowel tumors in capsule endoscopy frames using texture analysis based on the discrete wavelet transform. *Proceedings of the 30th Annual International Conference of the IEEE Engineering in Medicine and Biology Society (EMBS’08)*.

[20] Häfner M, Liedlgruber M, Uhl A, Vécsei A, Wrba F (2012). Delaunay triangulation-based pit density estimation for the classification of polyps in high-magnification chromo-colonoscopy. Comput. Methods Prog. Biomed.

[21] Gadermayr M, Uhl A (2014). Degradation adaptive texture classification. *Proceedings of the IEEE International Conference on Image Processing 2014 (ICIP’14)*.

[22] Gadermayr M, Hegenbart S, Uhl A (2014). Scale-adaptive texture classification. *Proceedings of 22nd IEEE International Conference on Pattern Recognition (ICPR’14)*.

[23] Hegenbart S, Uhl A, Vécsei A, Wimmer G (2013). Scale invariant texture descriptors for classifying celiac disease. Med. Image Anal.

[24] Zhang J, Tan T (2002). Brief review of invariant texture analysis methods. Pattern Recogn..

[25] Marziliano P, Dufaux F, Winkler S, Ebrahimi T, Sa G (2002). A no-reference perceptual blur metric. *Proceedings of the IEEE International Conference on Image Processing (ICIP’02)*.

[26] Crete F, Dolmiere T, Ladret P, Nicolas M (2007). The blur effect: perception and estimation with a new no-reference perceptual blur metric. *Proceedings of SPIE, Electronic Imaging Symposium Conf Human Vision and Electronic Imaging*.

[27] G Kylberg, The kylberg texture dataset v. 1.0. External report (Blue series) 35, Centre for Image Analysis, Swedish University of Agricultural Sciences and Uppsala University, Uppsala, Sweden (2011). http://www.cb.uu.se/~gustaf/texture/. Accessed 01 June 2014.

[28] Fan RE, Chang KW, Hsieh CJ, Wang XR, Lin CJ (2008). LIBLINEAR: a library for large linear classification. J. Mach. Learn. Res.

[29] Hegenbart S, Uhl A, Vécsei A (2011). Impact of endoscopic image degradations on LBP based features using one-class SVM for classification of celiac disease. *Proceedings of the 7th International Symposium on Image and Signal Processing and Analysis (ISPA’11)*.

[30] Liao S, Zhu X, Lei Z, Zhang L, Li S (2007). Learning multi-scale block local binary patterns for face recognition. *Advances in Biometrics*.

